# Development of cell labeling and gene editing tools in urochordate *Ciona*

**DOI:** 10.1007/s42995-025-00300-1

**Published:** 2025-06-03

**Authors:** Xiang Li, Lu Mu, Hongzhe Peng, Sun Nyunt Wai, Longjun Pu, Bo Dong

**Affiliations:** 1 Fang Zongxi Center for Marine EvoDevo, MoE Key Laboratory of Marine Genetics and Breeding, College of Marine Life Sciences, Ocean University of China, Qingdao, 266003 China; 2 Laboratory for Marine Biology and Biotechnology, Qingdao Marine Science and Technology Center, Qingdao, 266237 China; 3 Department of Molecular Biology, Umeå University, Umeå, Sweden; 4 Umeå Centre for Microbial Research (UCMR), Umeå University, Umeå, Sweden; 5 The Laboratory for Molecular Infection Medicine Sweden (MIMS), Umeå University, Umeå, Sweden; 6 Institute of Evolution and Marine Biodiversity, Ocean University of China, Qingdao, 266003 China

**Keywords:** *Ciona*, Gateway, Cell labeling, CRISPR/Cas9, Fluorescent sensor

## Abstract

Urochordate *Ciona* spp. are ideal marine model organisms for studying embryogenesis and developmental and evolutionary biology. However, the effective implementation of genetic labeling and CRISPR/Cas9-based editing tools at cellular resolution remains challenging. This study successfully developed and validated a collection of Gateway-based vectors for cell labeling in *Ciona* spp. The destination vector sets contained two Gateway cassettes flanked by Minos sites, allowing the N- or C-terminal tagging of a protein of interest with various fluorescent markers. In addition, we optimized the CRISPR/Cas9 and CRISPR/dCas9 systems by incorporating P2A-mCherry, a fluorescent indicator for Cas9 expression at cellular resolution. We demonstrated the effective destruction or inhibition of target genes when CRISPR constructs were introduced into fertilized eggs. Furthermore, we engineered a dual fluorescence sensor system that helps visualize successful gene knockouts at the cellular level in specific tissues. The genetic tools developed in this study offer a robust method for gene expression, cell tracking, and subcellular protein localization while also facilitating tissue-specific functional analysis in *Ciona* embryos and other model systems.

**Supplementary Information:**

The online version contains supplementary material available at 10.1007/s42995-025-00300-1.

## Introduction

The genus *Ciona*, belonging to the subphylum Urochordates, is an emerging, elegant model organism for developmental and cell biology studies. Key cellular processes during the early and late stages of *Ciona* embryogenesis, such as convergence and extension (Munro and Odell 2002), elongation (Lu et al. 2019; Sehring et al. 2014), and notochord tubulogenesis (Dong et al. 2009), have been characterized thoroughly. However, the underlying molecular mechanisms remain largely unknown, primarily due to the lack of effective genetic manipulation tools, which presents a significant technical barrier to systematically dissecting gene functions. The transgenic platform, which often requires the generation of various vectors, provides a powerful method for assessing gene expression and localizing proteins in *Ciona*. The recent utilization of recombination-based cloning systems in *Ciona*, particularly the Gateway technology, has remarkably streamlined vector construction. While such a set of Gateway compatible vectors has been developed (Roure et al. 2007), there is an urgent need for a platform that enables the systematic and multifunctional analyses of proteins of interest.

Over the past decade, the revolutionary gene editing tool CRISPR/Cas9 has been extensively employed to investigate gene functions across plants and animals (Barrangou and Doudna 2016; Pickar-Oliver and Gersbach 2019; Villiger et al. 2024). The development and application of the CRISPR/Cas9 system in *Ciona* have also been active, with several unique approaches reported for gene editing in this organism. The first method involved a ubiquitous gene knockout, which was achieved by either injecting the in vitro synthesized Cas9 mRNA and sgRNA or delivering the *Cr-Ef1α* > *Cas9* and *Cr-U6* > *sgRNA* plasmids into fertilized eggs via electroporation (Sasaki et al. 2014). An alternative approach employed a tissue-specific promoter to drive Cas9 expression, introduced into fertilized eggs by electroporation, leading to efficient tissue-specific mutations in *C*. *robusta* (Stolfi et al. 2014)*.* In addition, to achieve precise gene knock-in in *Ciona,* a strategy built upon a tissue-specific approach was developed by incorporating the DNA donor along with the Cas9-sgRNA complex (Pickett and Zeller 2018). Recent methods primarily involve ubiquitous gene knockout using Cas9/sgRNA ribonucleoprotein (RNP) microinjection (Reeves et al. 2021) and a more efficient editing approach employing the Cas9-Geminin fusion protein in *Ciona* (Pennati et al. 2024). Despite significant advancements achieved with these methods, accurately determining the Cas9 expression location and evaluating the gene editing efficiency at the cellular resolution remains challenging due to the mosaic expression of exogenous genes in *Ciona*. Another challenge in investigating gene functions in *Ciona* is the development of efficient gene knockdown methods, particularly when studying lethal genes or phenotypic hypomorphs. While dCas9 could potentially serve as an option, the tools necessary for this technique are currently unavailable in *Ciona*.

To address these gaps, we established a collection of Gateway vectors that enable tissue-specific expression of native or fusion proteins with diverse fluorescent tags, facilitating efficient cell labeling and systematic identification of localizing multiple proteins across various tissues and developmental stages in *Ciona*. In addition, we developed tools for gene knockout and knockdown, enabling cell-resolution precise genetic manipulation in a tissue-specific manner. We also introduced a novel system, Cas9-sensor, to accurately indicate gene editing events in *Ciona* cells. We anticipate these genetic tools to streamline gene expression, manipulation, and cell tracking, providing new opportunities to explore tissue-specific gene functions in *Ciona* embryos and other model systems.

## Materials and methods

### Animal collection, culture, and electroporation

Adult animals were collected from Rongcheng (Shandong Province, China) and acclimated under laboratory conditions at 16 ℃–20 ℃ for 1–2 days before the subsequent procedures. Fertilization and dechorionation followed the methods described previously (Christiaen et al. 2009). For electroporation, 300 μL of the dechorionated eggs were mixed with 80 μL of the plasmids or PCR products and 420 μL of the electroporation buffer (0.77 mol/L d-Mannitol in ddH_2_O/FSW at a 9:1 v/v ratio) in 4-mm cuvettes. The construct and DNA used in each electroporation are detailed in Supplementary Table S2. The electroporation was conducted using the exponential protocol: 50 V, 1500 μF, and timing resistor = ∞, on a Gene Pulser Xcell System (BIO-RAD, CA, USA). Subsequently, the fertilized eggs were washed once with FSW and then cultured at 16 ℃ or 18 ℃.

### Plasmid construction

The DNA fragments used for vector construction were either amplified using the Phanta Max Super-Fidelity DNA Polymerase (Nanjing Vazyme Biotech Co. Ltd., Nanjing, China) or obtained through the restriction digestion of existing plasmids. These DNA fragments were then purified using the GeneJET Gel Extraction Kit (Thermo Fisher, MA, USA) and ligated using the ClonExpress Ultra One Step Cloning Kit (Nanjing Vazyme, China).

The Gateway LR recombinational cloning reactions (Invitrogen, USA) were employed to generate the N- or C-terminal fusion expression vectors for constructing the Gateway expression clone.

To construct the sgRNA expression vectors, the empty sgRNA vector *Cr-U6* > *sgRNA(F* + *E)* (#59986; Addgene, MA, USA) or *hU6* > *sgRNA(F* + *E)* was linearized with *Bsa*I. The forward and reverse oligos were then ligated with the linearized vector using T4 ligase (TaKaRa Bio Inc., Shiga Japan). The plasmid* Cs-Brachyury* > *EGFP-Cs-Brachyury* > *mCherry* was linearized with *Bgl*II and *Hin*dIII for cloning the 20-bp sgRNA target sequence and the adjacent 3-bp PAM into the Cas9-sensor vector. It was followed by inserting the annealed oligos, Cr-ChM-G2-sensor-F1 and Cr-ChM-G2-sensor-R1, into the linearized vector with T4 ligase (TaKaRa, Japan).

For a comprehensive description of the complete sequences and the plasmid construction process, refer to the supplementary information (Supplementary Tables S3–S7). All plasmids were purified using either the EasyPure^®^Plasmid MiniPrep Kit (TransGen Biotech, Beijing, China) or the Genopure Plasmid Maxi Kit (Roche, Basel, Switzerland).

### Cell culture and transfection

The HEK293T cell line, kindly provided by Dr. Huarong Guo (Ocean University of China, Qingdao, China), was cultured in a high-glucose DMEM medium (VivaCell Biosciences, China) supplemented with 10% fetal bovine serum (FBS; VivaCell Biosciences, China) and 1% penicillin–streptomycin (Gibco, MA, USA). The cells were maintained under a 5% CO_2_ environment at 37 ℃ and were sub-cultured upon reaching 80%–90% confluency. Lipofectamine^™^ 3000 Transfection Reagent (Invitrogen, USA) was used for the transfection experiments.

### T7E1 cleavage assay

Embryos were lysed using PCR lysis buffer (20 mmol/L Tris–HCl, 100 mmol/L EDTA, 0.1% SDS, and 200 μg/mL Proteinase K in ddH_2_O; pH 8.0) at 60 ℃ for 10 min, followed by Proteinase K inactivation at 95 ℃ for 10 min. PCR was then performed using the lysate and Phanta Max Super-Fidelity DNA Polymerase (Nanjing Vazyme, China), with an annealing temperature of 55 ℃ or 60 ℃ for 15 s and an extension temperature of 72 °C for 30 s, for a total of 35 cycles. The amplicons were purified using the GeneJET Gel Extraction Kit (Thermo Fisher, USA). Next, 200 ng each of the mutant and wild-type amplicons (as controls) were diluted to 17 μL with ddH_2_O and mixed with 2 μL of 10 × T7 reaction buffer (Nanjing Vazyme, China) in a 200 μL PCR tube. Denaturation and annealing employed a thermal cycler, followed by incubation with 1 μL T7 Endonuclease I (Nanjing Vazyme, China) at 37 ℃ for 30 min. The products were then electrophoresed on a 2% agarose gel at 100 V for 30 min, and the gels were imaged and analyzed using ImageJ (https://imagej.net/ij/). The cleavage efficiency was determined as described before (Stolfi et al. 2014). Finally, the amplicons from the mutant gDNA were cloned into the *pEasy Blunt-3* vectors (TransGen Biotech, China) for Sanger sequencing (GENEWIZ, Suzhou, China).

### Immunofluorescence of *Ciona* embryos

*Ciona* embryos were collected at the desired developmental stage and fixed in 4% paraformaldehyde in filtered seawater (FSW) at room temperature for 2 h. The embryos were washed three times with 1 × PBS for 15 min each, followed by 0.1% Triton X-100 in 1 × PBS for 15 min. The embryos were blocked with the blocking buffer, 10% goat serum in PBST. After fixation and blocking, the embryos were incubated with an anti-Cr-ChM-1 primary antibody (Abmart, NJ, USA) diluted to 1:500 in the blocking buffer and stained overnight at 4 ℃. Subsequently, the samples were incubated with either Alexa 568-conjugated goat anti-rabbit IgG antibody (A-11036; Thermo Fisher) or Alexa Fluor 633-conjugated goat anti-rabbit IgG antibody (A-21070; Thermo Fisher), at 1:300 dilution in blocking buffer as the secondary antibody. Finally, the embryos were mounted in VECTASHIELD with DAPI (Vector Laboratories, CA, USA) and visualized with an A1 Plus confocal microscope (Nikon, Tokyo, China).

### Quantitative PCR

Total RNA was isolated using RNAiso Plus (Takara, Japan), and cDNA was synthesized using the HiScript II Q RT SuperMix for qPCR Kit (Nanjing Vazyme, China). Gene expression levels were ascertained using the ChamQ SYBR Color qPCR Master Mix (Nanjing Vazyme, China) on the LightCycler^®^ 480II System (Roche, Switzerland). Relative expression levels were calculated by applying the − 2^ΔΔCt^ method (Livak and Schmittgen 2001). The qPCR primers were designed with the Primer Premier 5 software (https://www.premierbiosoft.com/primerdesign/) and synthesized by the GENEWIZ Company (Suzhou, China). The primer sequences are listed in Supplementary Table S3.

### Confocal microscopy and 3D reconstruction

Confocal images were acquired using a TCS SP5 confocal laser scanning microscope (CLSM; Leica, Wetzlar, Germany) or an A1 plus confocal microscope (Nikon), equipped with 40 × oil and 63 × water immersion objectives; respective numerical apertures of 1.25 and 1.40. Z-series imaging was conducted at intervals of 1–1.2 µm, yielding image stacks composed of 20–40 images. Image analysis and 3D reconstruction were performed using the LAS AF software packages of the TCS SP5 system (Leica). In addition, the Adobe Photoshop software (https://www.adobe.com/) was employed for pseudocoloring.

### Statistical analysis

Quantitative data are presented as the means ± standard deviation (SD)/standard error (SEM). Inter-group comparative analyses were conducted by applying a two-tailed, unpaired Student’s* t* test. All data analyses were performed using Microsoft Excel 2019 (https://www.microsoft.com/). Statistical significance was determined at *P* < 0.05, with specific thresholds denoted as *P < 0.05, **P < 0.01, ***P < 0.001, and ****P < 0.0001.

## Results

### Establishment of a Gateway vector set for genetic labeling in *Ciona* cells

**Fig. 1:**
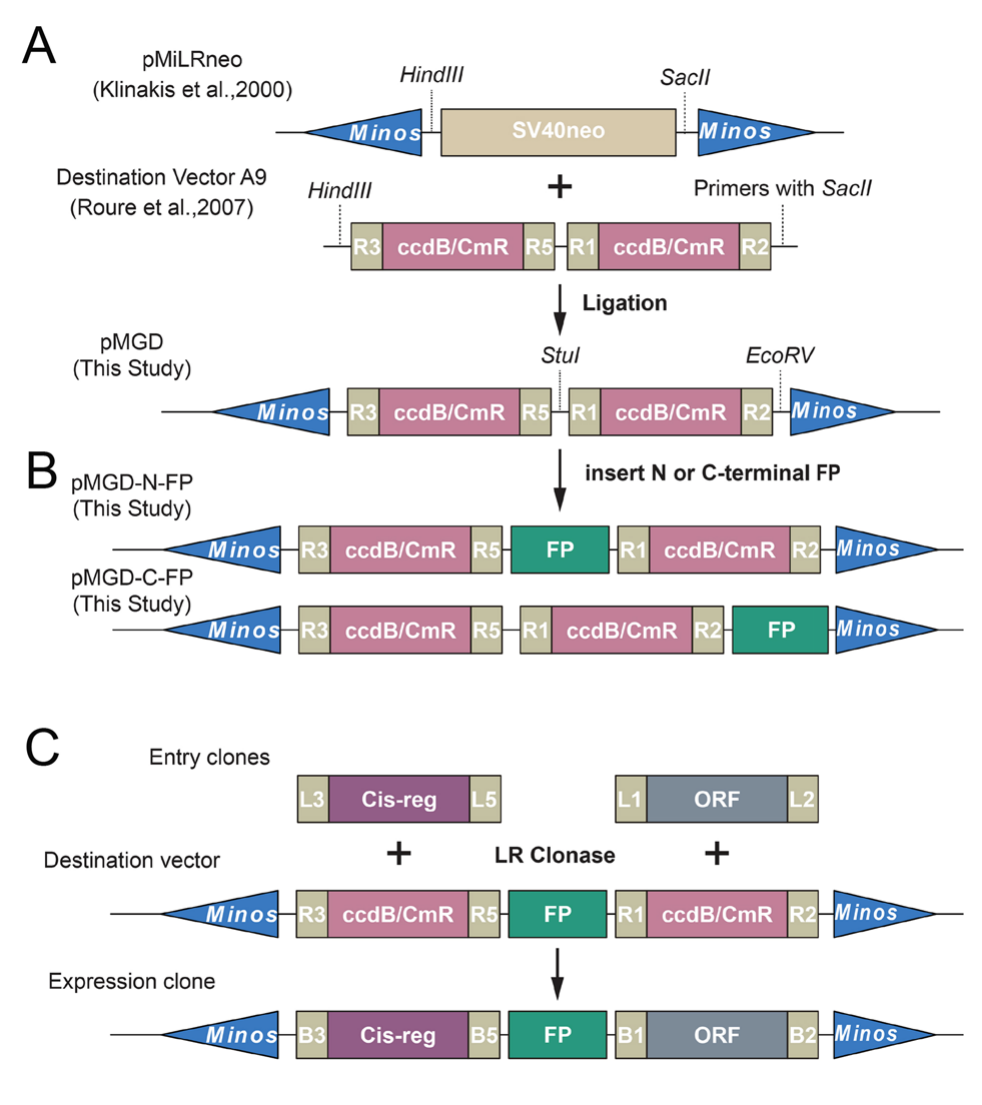
Scheme indicating the strategy for generating Gateway destination vectors and expression clones. **A** Construction of the primary Minos Gateway destination vector (*pMGD*) by replacing SV40neo in plasmid *pMiLRneo* with a fragment consisting of two Gateway cassettes from the destination vector A9 (*pSP1.72BSSPE-R3-ccdB/cmR-R5::RfA*). **B** Construction of the Gateway destination vector set (*pMGD-N-FP* or *pMGD-C-FP*) by cloning the N- or C-terminal fluorescent protein (FP) tags into the *pMGD* vector. **C** Generation of expression clones from our destination vector set by a streamlined one-step LR reaction

The Gateway system provides a highly efficient, compatible, and flexible method for directional recombination cloning. We aimed to create a versatile set of Gateway vectors suitable for genetic labeling in *Ciona*. The initial vector, termed *pMGD*, was generated from two existing plasmids: the destination vector *A9* (Roure et al. 2007) and *pMiLRneo* (Klinakis et al. 2000). It is distinguished by two Gateway cassettes flanked by Minos sites (Fig. 1A). For cell labeling, a fluorescent protein (FP) tag was inserted at either the N- or C-terminus of the second Gateway cassette, resulting in the final destination vector, *pMGD-N-FP* or *pMGD-C-FP* (Fig. 1B). Our vector set included a range of fluorescent tags, such as TagBFP, CFP, YFP, mVenus, GFP, mCherry, and mKate2. All destination vectors with these fluorescent tags are listed in Supplementary Table S1. Using the vector set, expression constructs with different fluorescent tags were efficiently and flexibly generated through a streamlined, one-step LR Gateway reaction by incorporating a cis-regulatory region and ORF entry clones into the first and second cassettes, respectively (Fig. 1C). These vectors provide an excellent platform for determining gene expression patterns and cell labeling, as well as for systematically visualizing multiple proteins of interest at the subcellular level.

### Verification of Gateway vectors in *Ciona* notochord cells

**Fig. 2:**
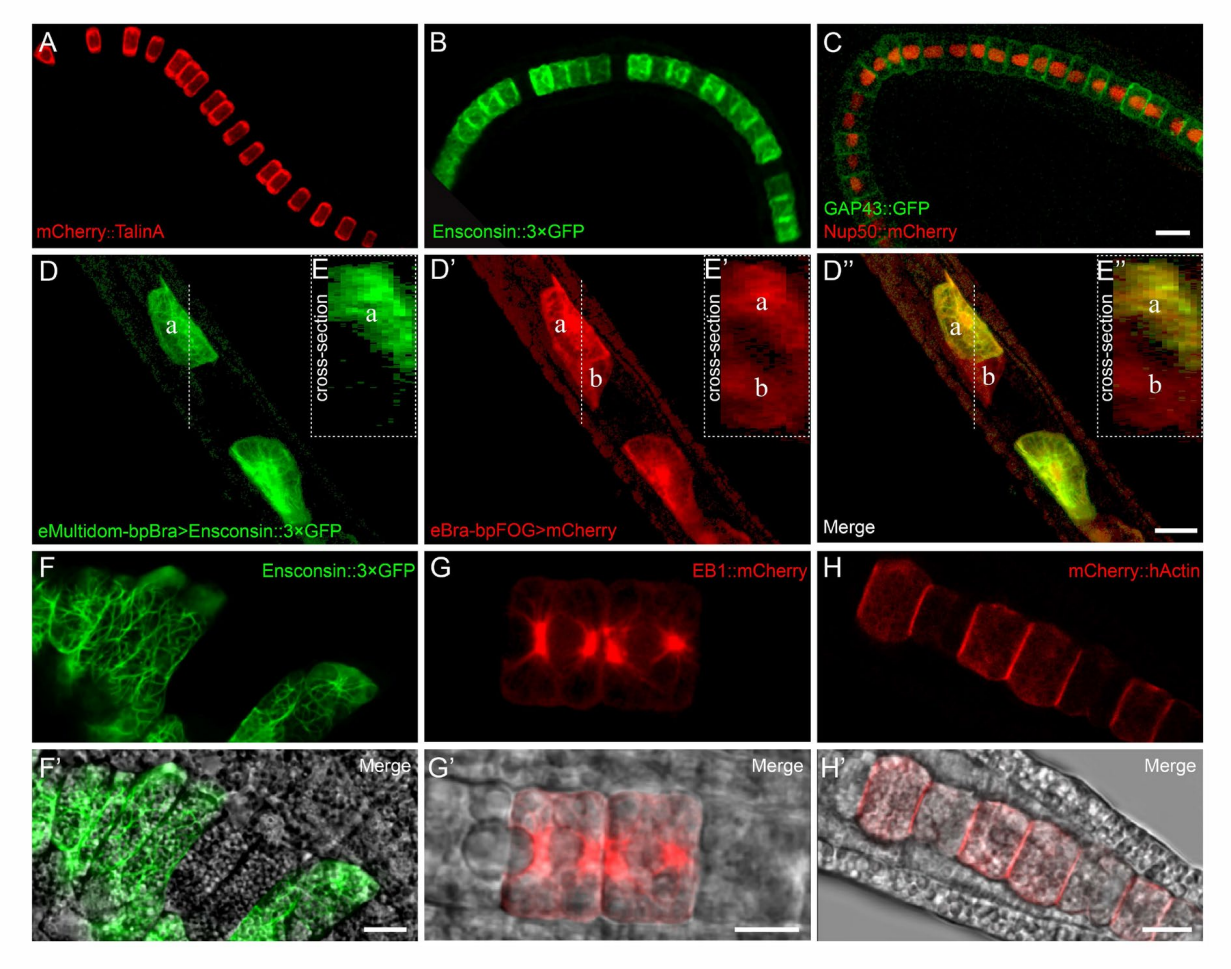
Labeling of the notochord cells of *Ciona robusta* with the Gateway vector set. **A**–**C** Representative images of notochord cells expressing clones *B3-eBra-bpFOG-B5* > *mCherry::B1-talinA-B2* (**A**), *B3-eBra-bpFOG-B5* > *B1-ensconsin::3* × *GFP-B2* (**B**), and *B3-eBra-bpFOG-B5* > *B1-GAP43-B2::GFP* together with *B3-eBra-bpFOG-B5* > *B1-Nup50-B2::mCherry* (**C**) in the late tailbud stage (stage 24) of *Ciona* embryos. The anterior is to the left. Scale bars: 50 μm. **D**–**D’’** and **E**–**E’’** Adjacent notochord cells marked with two expression clones driven by distinct notochord promoters in the hatching larval stage (stage 26) of *Ciona* embryos. *Ciona* embryos were co-electroporated with *B3-eMultidom-bpBra-B5* > *B1-ensconsin::3* × *GFP-B2* and *B3-eBra-bpFOG-B5* > *B1-B2::mCherry* expression clones. Cell “a” expresses GFP and mCherry simultaneously, whereas cell “b” expresses only mCherry. **D**, **D’**, and **D’’** show the 3D reconstruction of the notochord fragments. **E**, **E’**, and **E’’** show the cross-sections of the notochord cells at the planes indicated in (**D**, **D’**, and **D’’**), respectively. Scale bars: 10 μm. **F–H** and **F’**–**H’** Subcellular localization of the cytoskeletal proteins in *Ciona* notochord cells shown by the expression of *B3-eBra-bpFOG-B5* > *B1-ensconsin::3* × *GFP-B2* in (**F**), *B3-eBra-bpFOG-B5* > *B1-EB1-B2::mCherry* in (**G**) and *B3-eBra-bpFOG-B5* > *mCherry::B1-hActin-B2* in (**H**). **F’**–**H’** are merged images of notochord cells obtained by overlaying fluorescence images **F**–**H** onto bright field images. **F** was imaged at the early tailbud stage (stage 19), **G** was imaged at the late tailbud stage (stage 25), and **H** was imaged at the late tailbud stage (stage 24). Due to the mosaic expression in *Ciona* embryos, only some cells are labeled. Scale bars: 10 μm

The notochord cells in *Ciona* serve as a highly traceable model for imaging the spatial patterns of gene expression, prompting us to validate our Gateway system specifically in them. We generated diverse expression clones using our dual-cassette destination vectors by incorporating notochord-specific promoters and gene ORFs. Talin A, a cytoplasmic adapter, connects integrins to F-actin. We fused the I/LWEQ module of Talin A, harboring an actin-binding domain (Critchley 2009), with mCherry to construct the mCherry-TalinA vector; the fusion protein was localized to the cell cortex, just as Talin A (Fig. 2A). Ensconsin, a microtubule-binding protein (Roure et al. 2007), was tagged with a triple GFP (3 × GFP) to visualize the microtubule structure. When expressed in the notochord cells, Ensconsin-3 × GFP was enriched around the apical domain (Fig. 2B), where the extracellular lumen emerges quickly. Growth-associated protein 43 (GAP43), a membrane-associated phosphoprotein (Strittmatter et al. 1995), and Nup50, a component of the nuclear pore complex (Holzer and Antonin 2022), are widely used as cell membrane and nucleus markers in *Ciona*, respectively (Dong et al. 2009; Liang et al. 2024). Electroporating the *GAP43-GFP* and *Nup50-mCherry* mixture specifically labeled the cell boundaries and nuclei (Fig. 2C). As described, these four vectors expressed robustly with distinct spatial distributions in *Ciona* notochord cells, confirming the functionality of the Gateway vectors developed (Fig. 2A–C). Further, we successfully labeled adjacent notochord cells with GFP-tagged Ensconsin and cytosolic mCherry, driven by the respective promoters *eBra-bpFOG* and *eMultidom-bpBra*. As shown in Fig. 2D–D’’ and E–E’’, although both promoters demonstrated notochord cell-specific expression, the distinct mosaic patterns produced by them resulted in a unique spatial cell arrangement within the mature notochord tube, distinguishable by varied fluorescent colors. Furthermore, to validate the Gateway system at a subcellular resolution, we examined the localization of FP-tagged cytoskeletal proteins in detail. GFP-tagged Ensconsin and mCherry-tagged microtubule plus-end-tracking protein EB1 visualized the notochord microtubule filaments (Fig. 2F, F’, G, G’); human actin (hActin) fused to mCherry, successfully labeled F-actin bundles (Fig. 2H, H’). As expected, these proteins generated precise subcellular signals, effectively revealing the structures of different cytoskeletal systems (Fig. 2F–H and F’–H’). These findings suggest that the Gateway destination vector set established in this study is a powerful tool for cell labeling, either the same one or simultaneously different cells in *Ciona*. This system offers marked potential for analyzing the localization and interactions of proteins of interest at cellular resolution in animals with mosaic expression patterns.

### CRISPR/Cas9-based tools for gene editing at cellular resolution in *Ciona*

**Fig. 3:**
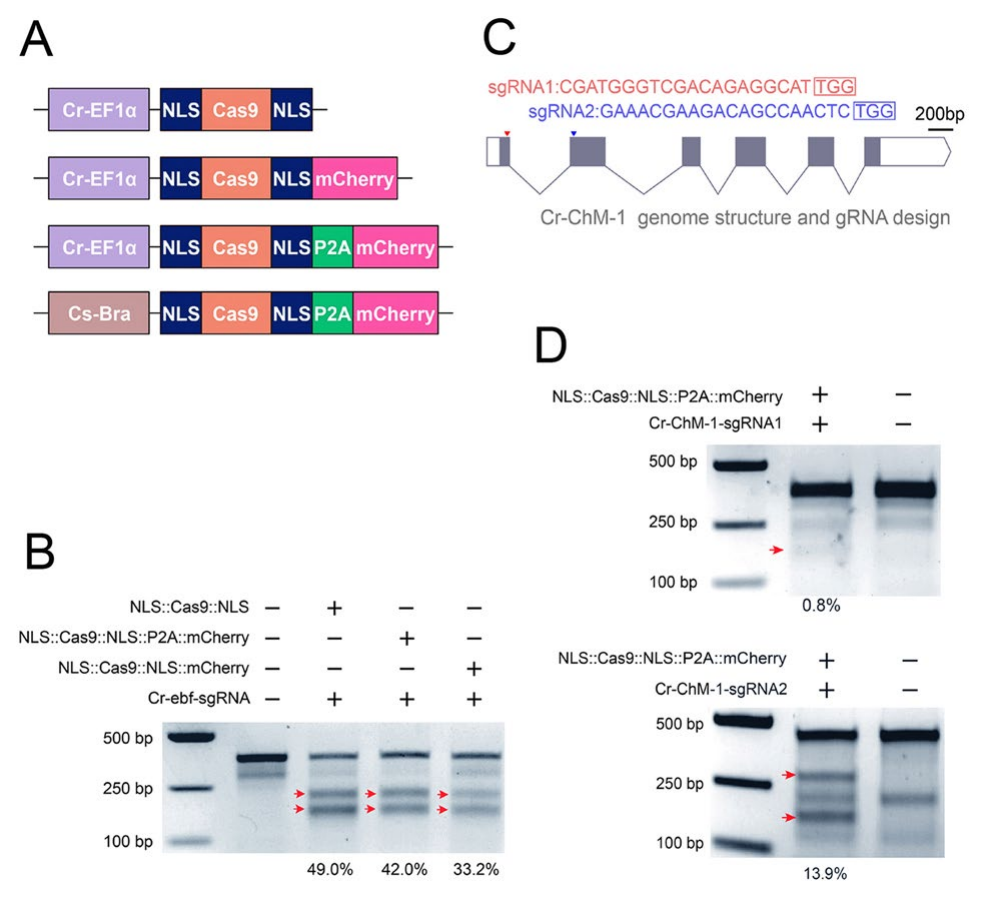
Development of Cas9 vectors and their evaluation for gene editing. **A** Cas9 constructs used in this study. The constructs *Cr-Ef1α* > *NLS::Cas9::NLS*, *Cr-Ef1α* > *NLS::Cas9::NLS::P2A::mCherry*, and *Cr-Ef1α* > *NLS::Cas9::NLS::mCherry* were designed for a ubiquitous gene knockout, and the construct *Cs*-*Bra* > *NLS::Cas9::NLS::P2A::mCherry* was designed for tissue-specific gene knockout*.* **B** Assessment of gene editing efficiency mediated by different Cas9 constructs using the T7 endonuclease I assay, targeting the *Cr-ebf* gene with sgRNAs expression driven by the U6 promoter of *Ciona robusta*. Red arrows indicate bands generated by T7E1 cleavage. For each group, 50 embryos were used. **C** *Cr-ChM-1* gene structure and sgRNA design. **D** Evaluation of the gene editing efficiency of two *Cr-ChM-1* sgRNAs using the T7 endonuclease I assay. The *Cr-Ef1α* > *NLS::Cas9::NLS* construct was used for Cas9 expression, with both sgRNAs driven by the U6 promoter of *C*. *robusta*. Red arrows indicate the bands generated by T7E1 cleavage. For each group, 50 embryos were used

The CRISPR/Cas9-based gene editing system has been successfully employed for gene disruption in the ascidian *Ciona* spp (Pickett and Zeller 2018; Sasaki et al. 2014; Stolfi et al. 2014). However, the inherent mosaic-patterned expression characteristics of *Ciona* pose a challenge in achieving precise gene knockout at a single-cell resolution. To overcome this problem, we developed an optimized approach by incorporating the FP-encoding *mCherry* into the original Cas9 constructs (*NLS::Cas9::NLS*) to create the *NLS::Cas9::NLS::mCherry* vector, which allows Cas9 expression visualization at the cellular level. In addition, to mitigate the potential impacts of mCherry fusion on Cas9 activity, a P2A self-cleaving peptide was inserted between the C-terminal NLS and the mCherry sequence, resulting in a further improved vector, *NLS::Cas9::NLS::P2A::mCherry* (Fig. 3A). We then assessed the gene knockout efficiency of each Cas9 construct by electroporating the fertilized *Ciona* eggs with the *Cr-ebf sgRNA* PCR product (Gandhi et al. 2017; Stolfi et al. 2014) and conducting the T7E1 assay. The data showed that the *NLS::Cas9::NLS* construct achieved the highest editing efficiency of 49.0%; *NLS::Cas9::NLS::P2A::mCherry* had a slightly lower efficiency of 42.0%; and *NLS::Cas9::NLS::mCherry* demonstrated the lowest efficiency at 33.2% (Fig. 3B). Based on these findings, the *NLS::Cas9::NLS::P2A::mCherry* construct was selected for further experiments.

**Fig. 4:**
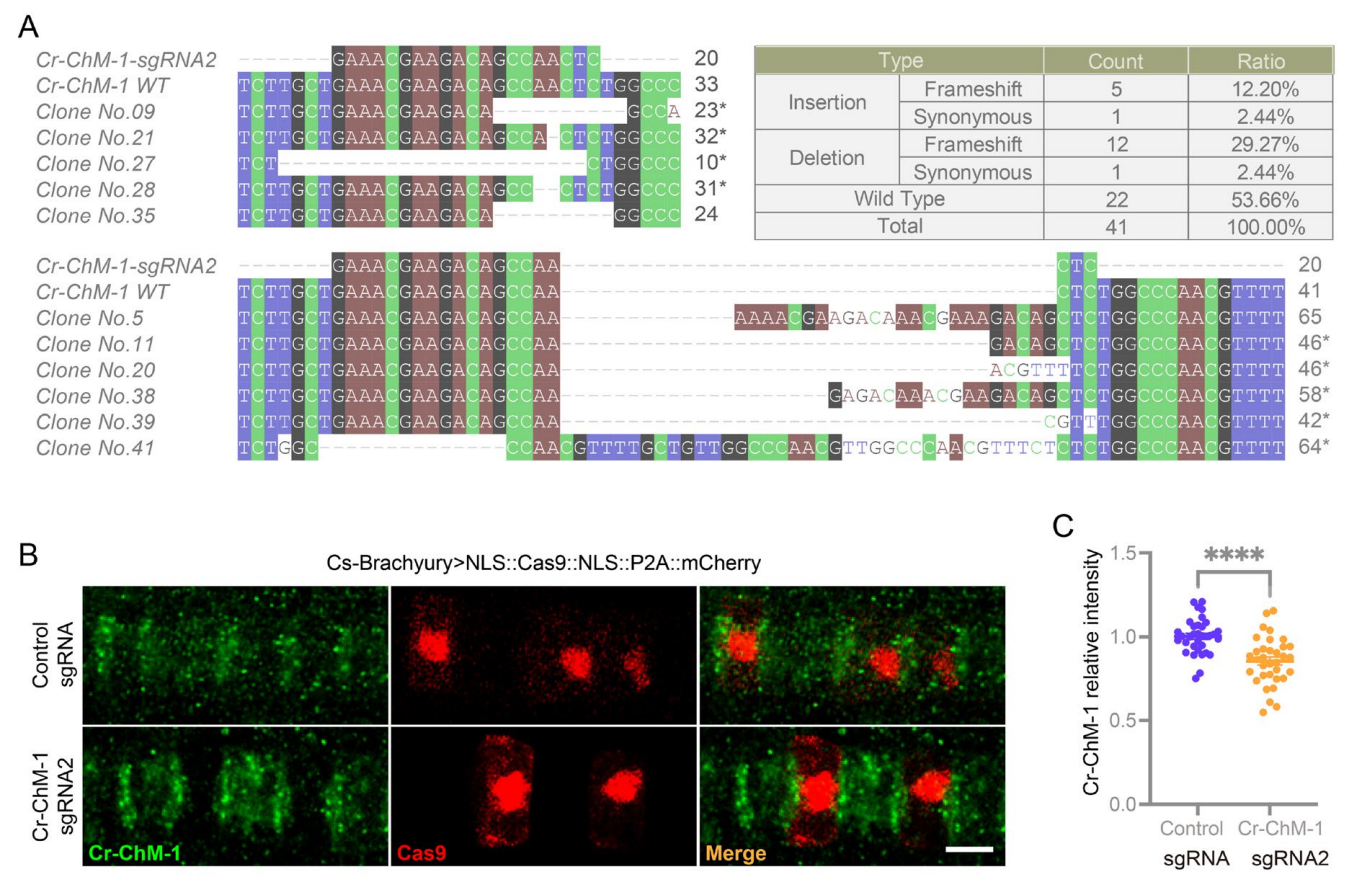
CRISPR/Cas9-based cell-resolution gene editing in *Ciona robusta*. **A** Mutation sequences and types introduced by Cas9/*Cr-ChM-1-sgRNA2*. * indicates the frameshift mutations. **B** Representative images showing the Cr-ChM-1 levels detected by immunofluorescence staining in notochord cells of the late tailbud stage (stage 24) of the embryos. Cr-ChM-1 levels were significantly reduced in the Cas9-positive notochord cells of the *Cr-ChM-1-sgRNA2* group. Scale bar: 10 μm. **C** Quantitative analysis of the relative intensity of Cr-ChM-1 in Cas9-positive notochord cells in the control and *Cr-ChM-1-sgRNA2* groups. Data are shown as the mean ± SEM. *n* = 34 for both groups. ****P < 0.0001; two-tailed *t* test

To evaluate the in vivo effectiveness of the *NLS::Cas9::NLS::P2A::mCherry* construct for gene editing at a cellular resolution, we targeted the *Ciona* notochord, focusing on the *Chondromodulin-1* (*Cr-ChM-1*) gene (Dou et al. 2018). For this, two single guide RNAs targeting different early exons, *Cr-ChM-1-sgRNA1* and *Cr-ChM-1-sgRNA2* (Fig. 3C), were designed using CRISPRdirect (http://crispr.dbcls.jp/) (Naito et al. 2015). The T7E1 assay revealed that *Cr-ChM-1-sgRNA1* had a low editing efficiency of ~ 0.8%, whereas *Cr-ChM-1-sgRNA2* demonstrated a markedly higher efficiency of ~ 13.9% (Fig. 3D). Further validation through Sanger sequencing indicated that 19 out of the 41 sequenced clones exhibited indel mutations (6 insertion and 13 deletion mutants) leading to protein frameshifts (Fig. 4A). Consequently, *Cr-ChM-1-sgRNA2* was selected for targeted gene knockout at the cellular level in *C*. *robusta*. For further investigations, the fertilized *Ciona* eggs were electroporated with the construct *Cs-Brachyury* > *NLS::Cas9::NLS::P2A::mCherry*, along with *Cr-ChM-1-sgRNA2* or a *control-sgRNA* PCR product. Immunofluorescence staining revealed a remarkable decline in Cr-ChM-1 expression in Cas9-mCherry^+^ notochord cells of the embryos introduced with *Cr-ChM-1-sgRNA2* compared to those with the control-sgRNA (Fig. 4B, C). These results demonstrate the effectiveness of our CRISPR/Cas9 tools for achieving cell-resolution gene editing in *Ciona.*

### Visualization of gene editing events in *Ciona* using the Cas9-sensor

**Fig. 5:**
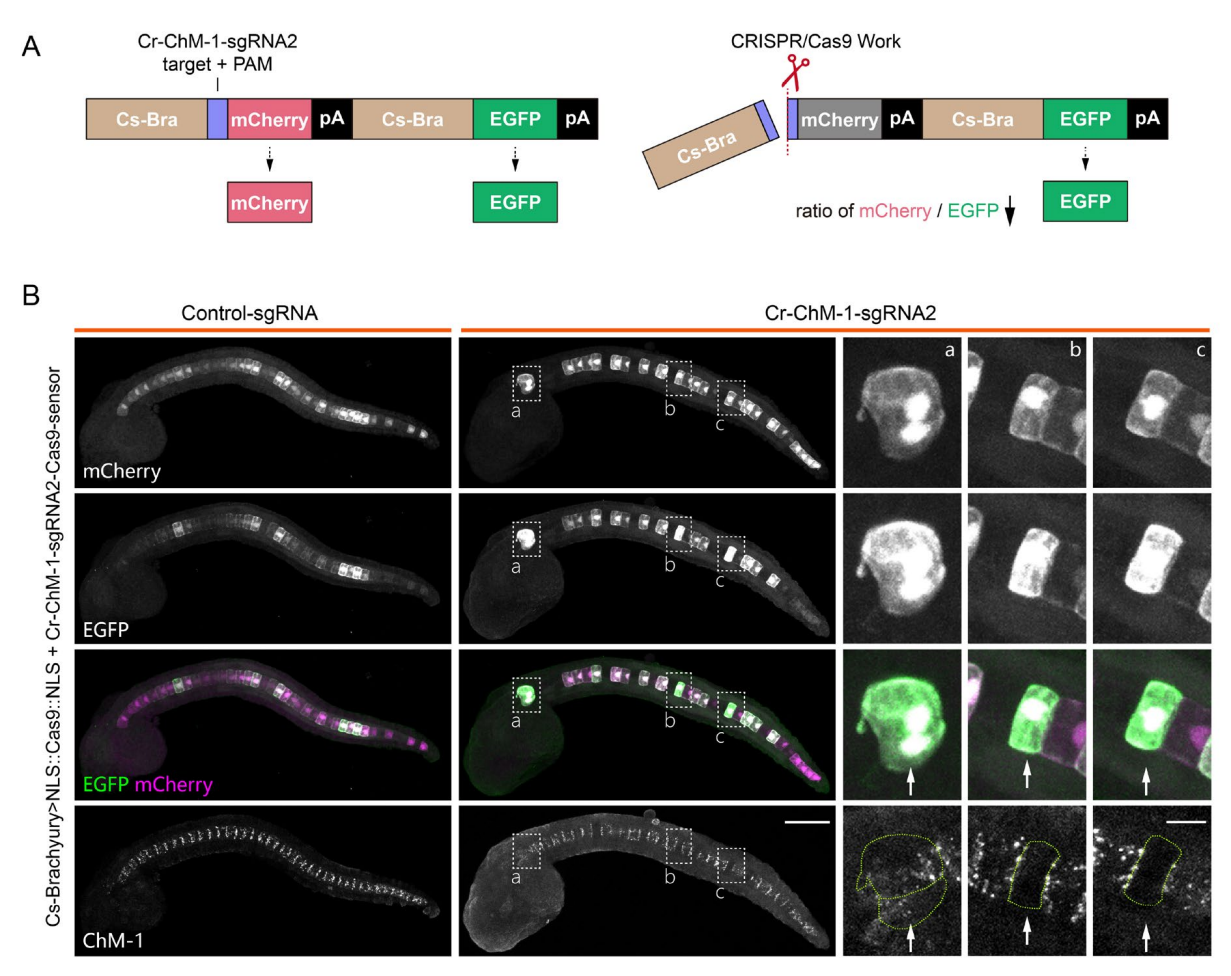
Visualization of gene editing events by the Cas9-sensor in notochord cells of *Ciona robusta.* **A** Cas9-sensor design for monitoring gene editing events. Successful cleavage of the target region disrupts mCherry expression, leaving EGFP unaffected and shifting toward green fluorescence. **B** Detection of Cr-ChM-1 disruption by the Cas9-sensor in notochord cells of the late tailbud stage (stage 24) in *Ciona* embryos. The notochord cells with enhanced green fluorescence ratios in the *Cr-ChM-1-sgRNA2* group are indicated by a, b, and c and outlined by a white dotted box. Scale bar: 50 μm. Enlarged cell images are shown on the right, indicated by white arrows, and cell boundaries are outlined by green dotted curves. Scale bar: 10 μm

The success of CRISPR/Cas9-mediated gene editing is influenced by numerous factors. These include efficient Cas9 expression, the co-presence of Cas9 protein and sgRNA within the same cell, and the on-target efficiency of the designed sgRNAs. Therefore, detecting Cas9 expression alone does not confirm a successful gene editing event. To address this issue, we developed a sensor system, termed the Cas9-sensor, designed to monitor gene editing events at the cellular level. The Cas9-sensor is based on the ratio of the fluorescence intensities produced by two reporters, both driven by the same but independent *Cs*-*brachyury* promoter within a single construct, to indicate the gene editing activity (Fig. 5A). Specifically, the sensor contains a 20-bp target sequence and an adjacent PAM site, both positioned upstream of the first reporter (mCherry). When the Cas9 protein successfully cleaves the target sequence and creates a DNA break, mCherry dissociates from its *Cs*-*brachyury* promoter, and its expression is disrupted; however, the second reporter, EGFP, continues to be expressed. This cleavage causes a shift to green fluorescence, indicating the occurrence of the gene editing event (Fig. 5A).

To validate this sensor system, we tested it in *Ciona* notochord cells during embryogenesis, utilizing the notochord-specific gene, *Cr-ChM-1*, as a target. We introduced the Cas9-expressing vector, the *Cr-ChM-1-sgRNA2* PCR product, and the sensor construct into fertilized eggs. As anticipated, cells with an enhanced ratio of green fluorescence displayed a marked reduction in Cr-ChM-1 expression, as confirmed by antibody staining in tailbud-stage embryos (Fig. 5B). These results demonstrate that our Cas9-sensor effectively serves as a reliable indicator of gene editing at the single-cell level in *Ciona* based on measuring the mCherry:EGFP ratio.

### CRISPR/dCas9-based tools for gene knockdown in *Ciona*

**Fig. 6:**
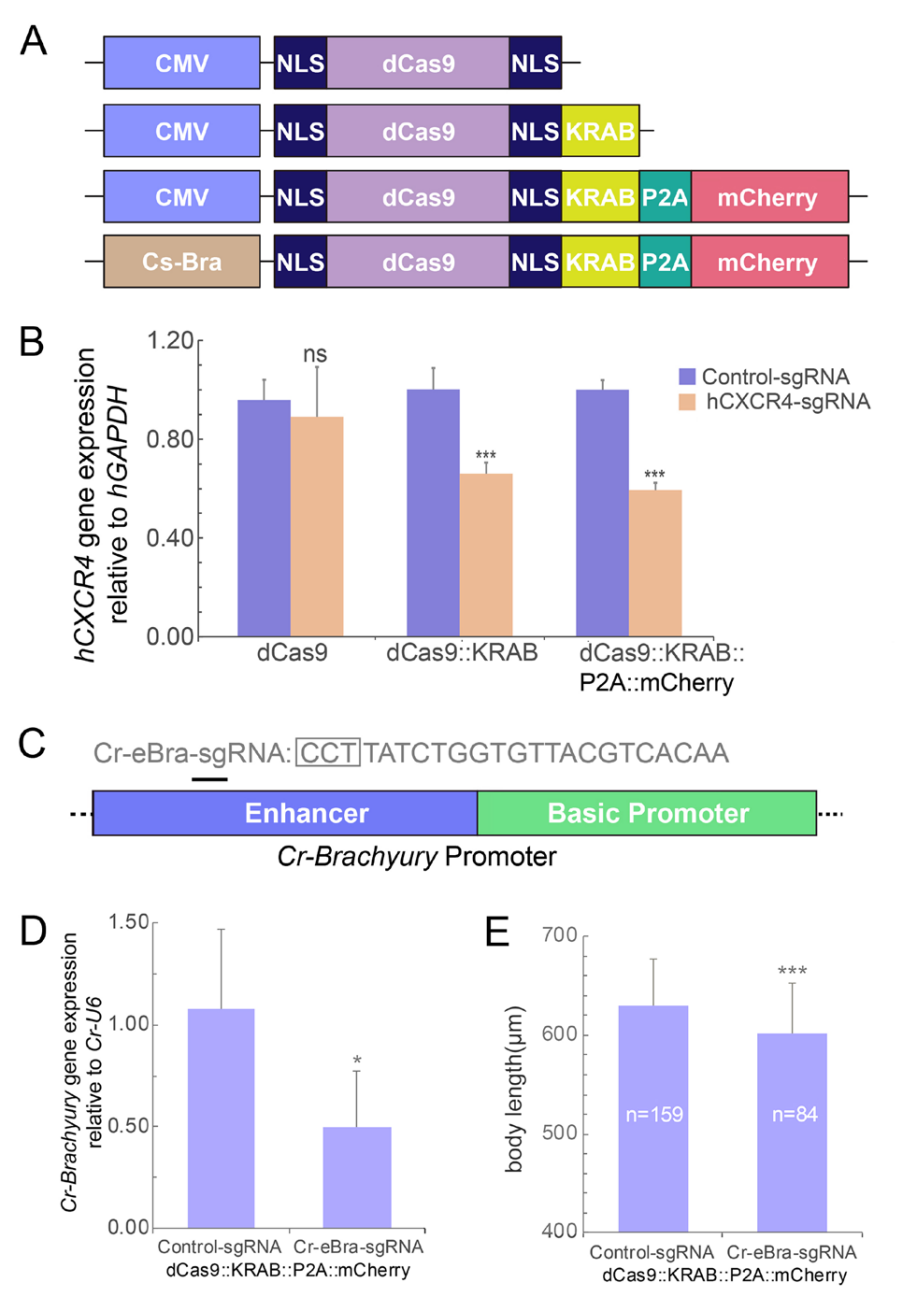
CRISPR/dCas9-based gene knockdown in *Ciona robusta.* **A** dCas9 constructs driven by the CMV and *Cs-brachyury* promoters for use in mammalian cells and *Ciona* notochord cells, respectively, used in this study. **B** Verification of gene knockdown mediated by different dCas9 constructs by targeting the *hCXCR4* gene in HEK293T cells. Data are presented as the mean ± SD, *n* = 3 biologic replicates. ***P < 0.001, ns: not significant, two-tailed *t* test. **C** *Cr-brachyury* promoter features and sgRNA design. **D** CRISPR/dCas9-KRAB-mediated *Cr-Brachyury* gene knockdown determined by qPCR using the construct *Cs*-*Bra* > *NLS::dCas9::NLS::KRAB::P2A::mCherry* with *Cr-eBra-sgRNA* expression driven by the U6 promoter of *C*. *robusta*. Data were obtained from late tailbud stage (stage 24) embryos and are shown as the mean ± SD; *n* = 3 biologic replicates. A total of 200 embryos were used in each group for each replicate. *P < 0.05, two-tailed *t* test. **E** Quantification of body length in the control and *Cr-Brachyury*-knockdown embryos. Data are shown as the mean ± SD; *n* = 159 for the control group and *n* = 84 for the *Cr-eBra-sgRNA* group. ***P < 0.001, two-tailed *t* test

While gene knockout is a definitive and heritable method for studying gene function, gene knockdown allows a more nuanced and flexible exploration without eliminating its expression. This approach is particularly valuable for investigating essential genes or when a more subtle modulation of gene expression is required. The dead Cas9 (dCas9) variant, a catalytically inactive form of Cas9, retains its capacity to bind to the target DNA but lacks the activity required for DNA cleavage (Doudna and Charpentier 2014). By fusing dCas9 with different functional domains, a range of molecular biology tools have been developed for gene knockdown (Gilbert et al. 2013, 2014; Qi et al. 2013), gene activation (Chavez et al. 2015; Gilbert et al. 2014; Jia et al. 2018; Prashant et al. 2013), and single-base editing (Ma et al. 2016). Fusing dCas9 with the transcriptional repression domain KRAB can remarkably impede gene expression in human cells (Gilbert et al. 2013). Based on these findings, we aimed to develop a gene knockdown tool in *Ciona*. To achieve this, we first created different versions of the dCas9 vectors driven by the CMV promoter, either with or without KRAB fusion and mCherry tagging, and tested their gene knockdown impacts in HEK293T cells (Fig. 6A). When the dCas9-KRAB fusion protein was coexpressed with a sgRNA targeting the human *CXCR4*, gene expression was inhibited more effectively compared with dCas9 alone (Fig. 6B).

**Fig. 7:**
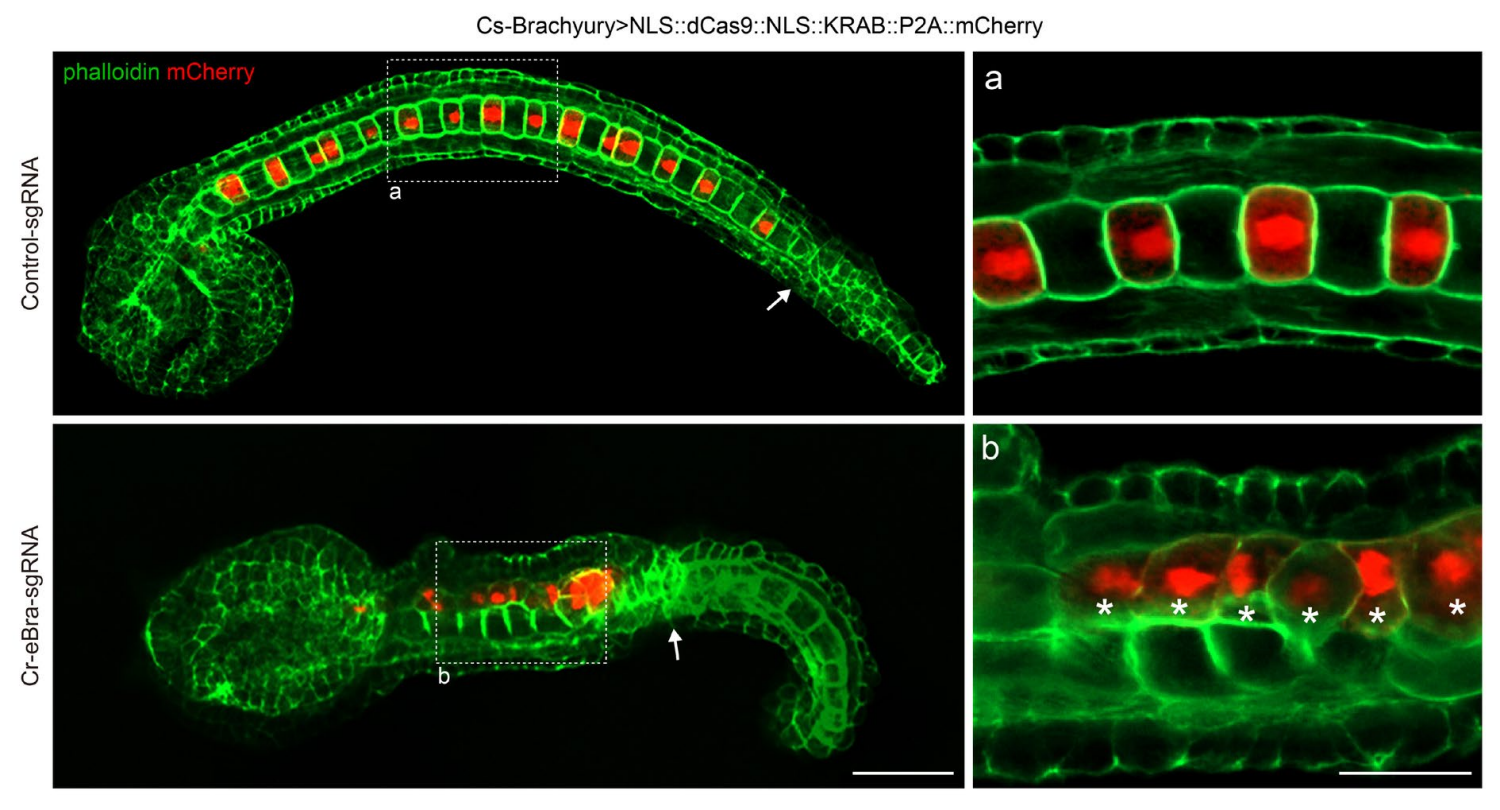
Gene knockdown induces morphologic changes in notochord cells of *Ciona robusta.* Representative images of the notochord cell morphology in the control and *Cr-Brachyury* knockdown animals mediated by dCas9-KRAB. All data were imaged at the late tailbud stage (stage 24). Scale bar: 50 μm. Areas “**a**” and “**b**” in the dashed box represent notochord sections with dCas9-KRAB expression. The epidermal cells are indicated by white arrows in the left panels. Enlarged images on the right highlight the potential knockdown cells indicated by white asterisks. Scale bar: 20 μm

Next, we explored the applicability of the dCas9-KRAB fusion protein for gene knockdown in *C*. *robusta* using the construct *Cs*-*Bra* > *NLS::dCas9::NLS::KRAB::P2A::mCherry*. Therefore, we selected *Brachyury*, a key transcription factor for notochord development, as our target, as its suppression results in notable and easily observable developmental defects, such as embryo elongation inhibition and notochord cell intercalation failure (Roure et al. 2007)*.* The minimal *Cr-Brachyury* promoter consists of a primary promoter and a 434-bp upstream element (Corbo et al. 1997). We designed a sgRNA, *Cr-eBra-sgRNA*, to target the upstream elements of the promoter (Fig. 6C). Upon examining *Cr*-*Brachyury* expression, we discovered that a combination of the dCas9-KRAB fusion protein and *Cr-eBra-sgRNA* caused significant gene inhibition in *C*. *robusta* (Fig. 6D)*.* In addition, the body lengths of the *Cr-Brachyury* knockdown embryos were noticeably shorter than the control group ones (Fig. 6E). Furthermore, cell morphology visualization revealed a marked suppression of notochord intercalation in *Cr-Brachyury* knockdown embryos (Fig. 7). Although some irregularly arranged, posterior cells were observed, they appeared to be epidermal rather than notochord cells due to varying thicknesses across embryos (Fig. 7). In summary, these results demonstrate that the dCas9-KRAB system enables effective gene knockdown in *Ciona*, highlighting its potential as a powerful tool for future dissections of gene function in this model organism.

## Discussion

The Gateway cloning system, developed by Invitrogen, MA, USA, is widely used for site-specific recombination in a variety of organisms, including *Caenorhabditis elegans* (Hope et al. 2004; Kogame 2015), *Drosophila* (Petersen and Stowers 2011), and zebrafish (Kemmler et al. 2023). This system enables the rapid exchange of large DNA fragments between different plasmids, utilizing two key enzyme-catalyzed reactions, BP and LR. In this study, we constructed and validated a multisite Gateway vector set featuring two Gateway cassettes designed specifically for entry clone recombination, flanked by Minos transposon sites. The set also offers a choice of various N- or C-terminal fluorescent tags for protein labeling. Our collection of constructs not only complements the existing Gateway vectors for *Ciona* (Roure et al. 2007) but also introduces several new functional features. First, our Gateway vector set enables the generation of fusion proteins with a wide array of fluorescent tags, such as TagBFP, CFP, YFP, mVenus, GFP, mCherry, and mKate2. These provide the opportunity to label multiple proteins and simultaneously visualize their cellular and subcellular locations. Moreover, the flexibility of fusing these tags with either the N- or C-terminus of the protein of interest minimizes the potential spatial effects of fusion, helping preserve the protein’s natural function and localization, thereby maintaining the results’ integrity. In addition, our vectors were equipped with two Minos transposon elements positioned on either side of the Gateway cassettes. Our experimental data confirmed that this configuration does not interfere with the expression or subcellular localization of the fused FPs in *Ciona* notochord cells. The Minos elements provide the potential to generate stable transgenic lines, not only in *Ciona* but also in other models, including *Drosophila*, mice, and human cell lines.

Labeling the neighboring cell boundaries is essential for investigating specific cellular processes, such as morphology, collective cell behaviors, and intercellular signal transduction. Fluorescent proteins are commonly employed for this purpose. However, co-introducing two FP-fusion constructs driven by the same promoter in *Ciona* often leads to identical mosaic expression patterns*.* This resulted in both fusion proteins being expressed in all positive cells, making it difficult to outline the cell boundaries distinctly. This phenomenon is likely due to the selective loss of transgenic DNA aggregates during the early cleavage stages, which causes simultaneous alterations in the expression of both transgenes (Zeller et al. 2006). Interestingly, we could label adjacent cells with distinct colors by utilizing constructs with varied regulatory elements, effectively overcoming this challenge. The current Gateway vector set provides a convenient and straightforward method to construct plasmids with different promoters, enabling the further exploration of this strategy to deepen our understanding of cellular processes. Beyond the advantages discussed here, our vector collection serves as an excellent basis for developing more innovative vectors. This ability opens up new avenues for a broader range of applications, such as bimolecular fluorescence complementation (BiFC) (Kim et al. 2007; Tian et al. 2011), fluorescence resonance energy transfer (FRET) (Mehlhorn et al. 2018; Wille et al. 2015), and yeast two-hybrid assays (Cao et al. 2011).

Although CRISPR/Cas9-based gene editing has been established in *Ciona* (Pickett and Zeller 2018; Reeves et al. 2021; Sasaki et al. 2014; Stolfi et al. 2014), the precise Cas9 functional site remains undefined due to the absence of suitable reporters and the occurrence of mosaic expression patterns, presenting a remarkable challenge for detecting gene manipulation at cellular resolution. The second part of this study was dedicated to developing traceable CRISPR-based genetic tools in *Ciona,* focusing on gene knockout and knockdown. We introduced the fluorescent reporter mCherry following the Cas9 protein, separated by a P2A self-cleaving peptide site, which is widely used in eukaryotic polycistronic expression (Donnelly et al. 2001; Ryan and Drew 1994; Trichas et al. 2008). This approach effectively monitored Cas9-expressing cells and identified gene editing events in the *Ciona* notochord system, thereby illustrating its potential for gene editing and gene function investigation at cellular resolution in a traceable manner. Furthermore, our study is the first to demonstrate that the dCas9-KRAB system can be utilized for gene suppression in *Ciona*. By applying the same design, i.e., incorporating the P2A site and mCherry after dCas9-KRAB, we successfully knocked down *Cr-Brachyury* and observed the cell phenotype, providing a novel method for dissecting gene function at the single-cell level. Compared with other methods, such as RNAi (Nishiyama and Fujiwara 2008), dCas9/KRAB-mediated gene knockdown possesses two distinct advantages. First, while RNAi uses the *Ciona* polymerase III promoter U6, resulting in the ubiquitous distribution of hairpin RNAs and lack of tissue specificity, dCas9-KRAB is driven by a polymerase II promoter, allowing for tissue-specific gene inhibition. Second, dCas9 provides greater scalability for developing innovative molecular biology tools. Beyond gene knockdown, dCas9 has wide applications, such as endogenous gene activation (Chavez et al. 2015; Gilbert et al. 2014), single-base editing (Ma et al. 2016; Rees and Liu 2018; Thuronyi et al. 2019), and prime editing (Anzalone et al. 2019). The gene knockdown approach established in this study lays a foundation for future advanced applications of dCas9 in *Ciona*.

However, the detection of Cas9 expression in a specific cell does not necessarily confirm a gene editing event, as it may be influenced by multiple factors. To address this limitation, we engineered a Cas9-sensor by integrating the target sequence with a dual fluorescence reporter, enabling the visualization of CRISPR/Cas9-mediated gene disruption at the single-cell level. During the experiment, the reporter construct was co-delivered with the CRISPR-associated plasmids, resulting in an extrachromosomal presentation of the target region, which differed significantly from its native chromosomal locus. This discrepancy means that even if a cleavage event is detected by our reporter system, as indicated by the reduced mCherry:EGFP intensity ratio, it does not confirm that the corresponding chromosomal locus in the same cell has also been edited. Additional methods, such as immunofluorescence staining with antibodies against the protein of interest, are necessary to confirm gene disruption.

Despite these limitations, the sensor developed here could greatly improve our real-time monitoring ability of the gene editing status, as demonstrated by our experimental data. In summary, our Gateway vector set and the genetic tools developed in this study represent a robust foundation for precise cell labeling and protein localization and offer powerful and versatile approaches to unravel gene function in *Ciona* and other organisms.

## Supplementary Information

Below is the link to the electronic supplementary material.

Supplementary file1 (XLSX 122 KB)

## Data Availability

All relevant data and resources can be found within the article and its supplementary information.

## References

[CR1] Anzalone AV, Randolph PB, Davis JR, Sousa AA, Koblan LW, Levy JM, Chen PJ, Wilson C, Newby GA, Raguram A, Liu DR (2019) Search-and-replace genome editing without double-strand breaks or donor DNA. Nature 576:149–157 31634902 10.1038/s41586-019-1711-4 PMC6907074

[CR2] Barrangou R, Doudna JA (2016) Applications of CRISPR technologies in research and beyond. Nat Biotechnol 34:933–941 27606440 10.1038/nbt.3659

[CR3] Cao S, Siriwardana CL, Kumimoto RW, Holt BF (2011) Construction of high quality Gateway™ entry libraries and their application to yeast two-hybrid for the monocot model plant *Brachypodium distachyon*. BMC Biotechnol 11:53 21595971 10.1186/1472-6750-11-53 PMC3239850

[CR4] Chavez A, Scheiman J, Vora S, Pruitt BW, Tuttle M, Iyer E, Lin S, Kiani S, Guzman CD, Wiegand DJ, Ter-Ovanesyan D, Braff JL, Davidsohn N, Housden BE, Perrimon N, Weiss R, Aach J, Collins JJ, Church GM (2015) Highly efficient Cas9-mediated transcriptional programming. Nat Methods 12:326–328 25730490 10.1038/nmeth.3312 PMC4393883

[CR5] Christiaen L, Wagner E, Shi W, Levine M (2009) Isolation of sea squirt (*Ciona*) gametes, fertilization, dechorionation, and development. CSH Protoc 2009:pdb.prot5344 10.1101/pdb.prot5344 20150091

[CR6] Corbo JC, Levine M, Zeller RW (1997) Characterization of a notochord-specific enhancer from the Brachyury promoter region of the ascidian, *Ciona intestinalis*. Development 124:589–602 9043074 10.1242/dev.124.3.589

[CR7] Critchley DR (2009) Biochemical and structural properties of the integrin-associated cytoskeletal protein talin. Ann Rev Biophys 38:235–254 19416068 10.1146/annurev.biophys.050708.133744

[CR8] Dong B, Horie T, Denker E, Kusakabe TG, Tsuda M, Smith WC, Jiang D (2009) Tube formation by complex cellular processes in *Ciona intestinalis* notochord. Dev Biol 330:237–249 19324030 10.1016/j.ydbio.2009.03.015 PMC2841060

[CR9] Donnelly MLL, Hughes LE, Luke G, Mendoza H, Dam ET, Gani D, Ryan MD (2001) The ‘cleavage’ activities of foot-and-mouth disease virus 2A site-directed mutants and naturally occurring ‘2A-like’ sequences. J Gen Virol 82:1027–1041 11297677 10.1099/0022-1317-82-5-1027

[CR10] Dou X, Xiang L, Haiyan Y, Bo D (2018) Dual roles of ascidian chondromodulin-1: promoting cell proliferation whilst suppressing the growth of tumor cells. Mar Drugs 16:59 29439497 10.3390/md16020059 PMC5852487

[CR11] Doudna JA, Charpentier E (2014) The new frontier of genome engineering with CRISPR-Cas9. Science 346:1258096 25430774 10.1126/science.1258096

[CR12] Gandhi S, Haeussler M, Razykrajka F, Christiaen L, Stolfi A (2017) Evaluation and rational design of guide RNAs for efficient CRISPR/Cas9-mediated mutagenesis in *Ciona*. Dev Biol 425:8–20 28341547 10.1016/j.ydbio.2017.03.003 PMC5502750

[CR13] Gilbert LA, Larson MH, Morsut L, Liu Z, Brar GA, Torres SE, Stern-Ginossar N, Brandman O, Whitehead EH, Doudna JA, Lim WA, Weissman JS, Qi LS (2013) CRISPR-mediated modular RNA-guided regulation of transcription in eukaryotes. Cell 154:442–451 23849981 10.1016/j.cell.2013.06.044 PMC3770145

[CR14] Gilbert LA, Horlbeck MA, Adamson B, Villalta JE, Chen Y, Whitehead EH, Guimaraes C, Panning B, Ploegh HL, Bassik MC, Qi LS, Kampmann M, Weissman JS (2014) Genome-scale CRISPR-mediated control of gene repression and activation. Cell 159:647–661 25307932 10.1016/j.cell.2014.09.029 PMC4253859

[CR15] Holzer G, Antonin W (2022) Nup50 plays more than one instrument. Cell Cycle 21:1785–1794 35549614 10.1080/15384101.2022.2074742 PMC9359400

[CR16] Hope IA, Stevens J, Garner A, Hayes J, Cheo DL, Brasch MA, Vidal M (2004) Feasibility of genome-scale construction of promoter::reporter gene fusions for expression in *Caenorhabditis elegans* using a multisite gateway recombination system. Genome Res 14:2070–2075 15489328 10.1101/gr.2463804 PMC528922

[CR17] Jia Y, Xu RG, Ren X, Ewen-Campen B, Rajakumar R, Zirin J, Yang-Zhou D, Zhu R, Wang F, Mao D (2018) Next-generation CRISPR/Cas9 transcriptional activation in *Drosophila* using flySAM. Proc Natl Acad Sci USA 115:4719–4724 29666231 10.1073/pnas.1800677115 PMC5939105

[CR18] Kemmler CL, Moran HR, Murray BF, Scoresby A, Klem JR, Eckert RL, Lepovsky E, Bertho S, Nieuwenhuize S, Burger S, D’Agati G, Betz C, Puller AC, Felker A, Ditrychova K, Botschi S, Affolter M, Rohner N, Lovely CB, Kwan KM et al (2023) Next-generation plasmids for transgenesis in zebrafish and beyond. Development 150:201531 10.1242/dev.201531 PMC10263156 36975217

[CR19] Kim M-H, Roh H-E, Lee M-N, Hur M-WJCP (2007) New fast BiFC plasmid assay system for *in vivo* protein-protein interactions. Cell Physiol Biochem 20:703–714 17982253 10.1159/000110431

[CR20] Klinakis AG, Loukeris TG, Pavlopoulos A, Savakis C (2000) Mobility assays confirm the broad host-range activity of the *Minos* transposable element and validate new transformation tools. Insect Mol Biol 9:269–275 10886410 10.1046/j.1365-2583.2000.00183.x

[CR21] Kogame T (2015) 4-Fragment Gateway cloning format for MosSCI-compatible vectors integrating promoterome and 3′UTRome libraries of *Caenorhabditis elegans*. J Med Invest 62:161–166 26399341 10.2152/jmi.62.161

[CR22] Liang Z, Dondorp DC, Chatzigeorgiou M (2024) The ion channel Anoctamin 10/TMEM16K coordinates organ morphogenesis across scales in the urochordate notochord. PLoS Biol 22:e3002762 39173068 10.1371/journal.pbio.3002762 PMC11341064

[CR23] Livak KJ, Schmittgen TD (2001) Analysis of relative gene expression data using real-time quantitative PCR and the 2^−ΔΔCt^ method. Methods 25:402–408 11846609 10.1006/meth.2001.1262

[CR24] Lu Q, Bhattachan P, Dong B (2019) Ascidian notochord elongation. Dev Biol 448:147–153 30458170 10.1016/j.ydbio.2018.11.009

[CR25] Ma Y, Zhang J, Yin W, Zhang Z, Song Y, Chang X (2016) Targeted AID-mediated mutagenesis (TAM) enables efficient genomic diversification in mammalian cells. Nat Methods 13:1029 27723754 10.1038/nmeth.4027

[CR26] Mehlhorn DG, Wallmeroth N, Berendzen KW, Grefen C (2018) 2in1 vectors improve in planta BiFC and FRET analyses. In: Hawes C, Kriechbaumer V (eds) The plant endoplasmic reticulum. Methods in molecular biology, vol 1691. Humana Press, New York, NY, pp 139–158 10.1007/978-1-4939-7389-7_11 29043675

[CR27] Munro E, Odell GM (2002) Polarized basolateral cell motility underlies invagination and convergent extension of the ascidian notochord. Development 129:13–24 11782397 10.1242/dev.129.1.13

[CR28] Naito Y, Hino K, Bono H, Ui-Tei K (2015) CRISPRdirect: software for designing CRISPR/Cas guide RNA with reduced off-target sites. Bioinformatics 31:1120–1123 25414360 10.1093/bioinformatics/btu743 PMC4382898

[CR29] Nishiyama A, Fujiwara S (2008) RNA interference by expressing short hairpin RNA in the *Ciona intestinalis* embryo. Dev Growth Differ 50:521–529 18510713 10.1111/j.1440-169X.2008.01039.x

[CR30] Pennati A, Jakobi M, Zeng F, Ciampa L, Rothbächer U (2024) Optimizing CRISPR/Cas9 approaches in the polymorphic tunicate *Ciona intestinalis*. Dev Biol 510:31–39 38490564 10.1016/j.ydbio.2024.03.003

[CR31] Petersen LK, Stowers RS (2011) A Gateway MultiSite recombination cloning toolkit. PLoS ONE 6:e24531 21931740 10.1371/journal.pone.0024531 PMC3170369

[CR32] Pickar-Oliver A, Gersbach CA (2019) The next generation of CRISPR-Cas technologies and applications. Nat Rev Mol Cell Bio 20:490–507 31147612 10.1038/s41580-019-0131-5 PMC7079207

[CR33] Pickett CJ, Zeller RW (2018) Efficient genome editing using CRISPR-Cas-mediated homology directed repair in the ascidian *Ciona robusta*. Genesis 56:e23260 30375719 10.1002/dvg.23260 PMC6312475

[CR34] Prashant M, John A, Benjamin SP, Esvelt KM, Mark M, Sriram K, Luhan Y, Church GM (2013) Cas9 transcriptional activators for target specificity screening and paired nickases for cooperative genome engineering. Nat Biotechnol 31:833–838 23907171 10.1038/nbt.2675 PMC3818127

[CR35] Qi LS, Larson MH, Gilbert LA, Doudna JA, Weissman JS, Arkin AP, Lim WA (2013) Repurposing CRISPR as an RNA-guided platform for sequence-specific control of gene expression. Cell 152:1173–1183 23452860 10.1016/j.cell.2013.02.022 PMC3664290

[CR36] Rees HA, Liu DR (2018) Base editing: precision chemistry on the genome and transcriptome of living cells. Nat Rev Genet 19:770–788 30323312 10.1038/s41576-018-0059-1 PMC6535181

[CR37] Reeves WM, Shimai K, Winkley KM, Veeman MT (2021) Brachyury controls *Ciona* notochord fate as part of a feedforward network. Development 148:dev195230 33419874 10.1242/dev.195230 PMC7875503

[CR38] Roure A, Rothbacher U, Robin F, Kalmar E, Ferone G, Lamy C, Missero C, Mueller F, Lemaire P (2007) A multicassette gateway vector set for high throughput and comparative analyses in *Ciona* and vertebrate embryos. PLoS ONE 2:e916 17878951 10.1371/journal.pone.0000916 PMC1976267

[CR39] Ryan MD, Drew J (1994) Foot-and-mouth disease virus 2A oligopeptide mediated cleavage of an artificial polyprotein. EMBO J 13:928–933 8112307 10.1002/j.1460-2075.1994.tb06337.x PMC394894

[CR40] Sasaki H, Yoshida K, Hozumi A, Sasakura Y (2014) CRISPR/Cas9-mediated gene knockout in the ascidian *Ciona intestinalis*. Dev Growth Differ 56:499–510 25212715 10.1111/dgd.12149 PMC4231237

[CR41] Sehring IM, Dong B, Denker E, Bhattachan P, Deng W, Mathiesen BT, Jiang D (2014) An equatorial contractile mechanism drives cell elongation but not cell division. PLoS Biol 12:e1001781 24503569 10.1371/journal.pbio.1001781 PMC3913557

[CR42] Stolfi A, Gandhi S, Salek F, Christiaen L (2014) Tissue-specific genome editing in *Ciona* embryos by CRISPR/Cas9. Development 141:4115–4120 25336740 10.1242/dev.114488 PMC4302896

[CR43] Strittmatter SM, Fankhauser C, Huang PL, Mashimo H, Fishman MC (1995) Neuronal pathfinding is abnormal in mice lacking the neuronal growth cone protein GAP-43. Cell 80:445–452 7859286 10.1016/0092-8674(95)90495-6

[CR44] Thuronyi BW, Koblan LW, Levy JM, Yeh W, Zheng C, Newby GA, Wilson C, Bhaumik M, Shubinaoleinik O, Holt JR, Liu DR (2019) Continuous evolution of base editors with expanded target compatibility and improved activity. Nat Biotechnol 37:1070–1079 31332326 10.1038/s41587-019-0193-0 PMC6728210

[CR45] Tian G, Lu Q, Zhang L, Kohalmi SE, Cui Y (2011) Detection of protein interactions in plant using a gateway compatible bimolecular fluorescence complementation (BiFC) system. J Vis Exp 55:e3473 10.3791/3473 PMC3230203 21947026

[CR46] Trichas G, Begbie J, Srinivas S (2008) Use of the viral 2A peptide for bicistronic expression in transgenic mice. BMC Biol 6:40 18793381 10.1186/1741-7007-6-40 PMC2553761

[CR47] Villiger L, Joung J, Koblan L, Weissman J, Abudayyeh OO, Gootenberg JS (2024) CRISPR technologies for genome, epigenome and transcriptome editing. Nat Rev Mol Cell Bio 25:464–487 38308006 10.1038/s41580-023-00697-6

[CR48] Wille T, Barlag B, Jakovljevic V, Hensel M, Sourjik V, Gerlach RG (2015) A gateway-based system for fast evaluation of protein-protein interactions in bacteria. PLoS ONE 10:e0123646 25856398 10.1371/journal.pone.0123646 PMC4391838

[CR49] Zeller RW, Weldon DS, Pellatiro MA, Cone AC (2006) Optimized green fluorescent protein variants provide improved single cell resolution of transgene expression in ascidian embryos. Dev Dyn 235:456–546 16287050 10.1002/dvdy.20644

